# Efficacy of two different dosages of levofloxacin in curing Helicobacter pylori infection: A Prospective, Single-Center, randomized clinical trial

**DOI:** 10.1038/s41598-018-27482-2

**Published:** 2018-06-13

**Authors:** Huo-Ye Gan, Tie-Li Peng, You-Ming Huang, Kai-Hua Su, Lin-Li Zhao, Li-Ya Yao, Rong-Jiao Yang

**Affiliations:** Department of Gastroenterology, The Sixth Affiliated Hospital of Guangzhou Medical University, Qingyuan People’s Hospital, Guangdong, China

## Abstract

Bismuth + proton pump inhibitor (PPI) + amoxicillin + levofloxacin is one of the bismuth quadruple therapy regimens widely used for the eradication of *H. pylori* infection. The recommended dosage of levofloxacin is 500 mg once daily or 200 mg twice daily to eradicate *H. pylori* infection. The aim of the present open-label, randomized control trial was to compare the effectiveness, safety, and compliance of different dosages of levofloxacin used to cure *Helicobacter pylori* infection. Eligible patients were randomly assigned to receive esomeprazole, amoxicillin, colloidal bismuth pectin and levofloxacin 500 mg once/day (group A) or levofloxacin 200 mg twice/day (group B) for 14 days. The primary outcome was the eradication rates in the intention-to-treat (ITT) and per protocol (PP) analyses. Overall, 400 patients were enrolled. The eradication rates in group A and group B were 77.5% and 79.5% respectively, in the ITT analysis, and 82.9% and 86.4%, respectively, in the PP analysis. No significant differences were found between two groups in terms of eradication rate, adverse effects or compliance. Oral levofloxacin 200 mg twice daily was similar in efficacy for eradicating *H. pylori* infection to oral levofloxacin 500 mg once daily but with lower mean total costs.

## Introduction

Epidemiological studies have indicated that the prevalence of *Helicobacter pylori* (*H. pylori*) infection has an incidence of 20–50% in developed countries and 40–60% in China^1,2^. *H. pylori* plays a causative role in many diseases, including gastritis, peptic ulcers, gastric mucosa-associated lymphoid tissue (MALT) lymphoma and gastric cancer^3–8^. Eradication of *H. pylori* infection should be recommended to *H. pylori*-infected patients unless they have competing considerations^9^.

Drug resistance in many countries has increased^10–13^. The drug resistance rates against metronidazole, clarithromycin, and levofloxacin have increased substantially in many regions. In China, the rates of resistance to metronidazole, clarithromycin, and levofloxacin are 60–70%, 20–38%, and 30–38%, respectively^14–16^. The eradication rate of standard triple therapy has fallen to lower or far lower than 80% in many countries because of the drug resistance rate of *H. pylori*^17^. The Maastricht V/Florence Consensus Report recommended bismuth quadruple therapy (BQT) as the first-line treatment for the eradication of *H. pylori* infection in areas of high dual clarithromycin and metronidazole resistance^18^. The Fourth Chinese National Consensus Report on the management of *H. pylori* infection recommended that bismuth + proton pump inhibitor (PPI) + amoxicillin + levofloxacin could be one of the BQT regimens used as a first-line treatment for the eradication of *H. pylori* infection. The recommended dosage of levofloxacin was 500 mg once daily or 200 mg twice daily. To evaluate the effect of these two different dosages of levofloxacin for the cure of *H. pylori* infection, we conducted a prospective, randomized control trial. All authors had access to the study data and reviewed and approved the final manuscript.

## Materials and Methods

### Study Design

This was a prospective, randomized control, single-center clinical study. This study was performed in The Sixth Affiliated Hospital of Guangzhou Medical University (Qingyuan People’s Hospital) between April and September 2017. The patients with dyspepsia referred for an upper endoscopy and diagnosed with chronic gastritis with dyspepsia or mucosal atrophy/erosion were eligible for enrollment if they had an *H. pylori* infection, were 20–60 years old, and had never previously received eradication treatment for *H. pylori* infection. Patients who met one of the following criteria were excluded from the study: patients taking any medicine that could influence the study results, such as antibiotics, bismuth salts, PPIs or histamine-2-receptor blockers in the previous 4 weeks; patients with peptic ulcers, gastrointestinal malignancies, previous gastric or esophageal surgery, or severe concomitant diseases; patients with histories of allergies to any of the study medicines; patients who were currently pregnant or lactating; and patients who could not give informed consent by themselves or who refused to participate in the trial. This study was approved by the Institutional Review Board of The Sixth Affiliated Hospital of Guangzhou Medical University. Each patient provided written informed consent before beginning the study procedure. The design and procedures of the study were carried out in accordance with the principles of the Declaration of Helsinki. This study was registered in the Chinese Clinical Trial Registry on13/01/2017 (clinical trial registration number: ChiCTR-IPD-17010408).

### Intervention

Eligible patients were randomly divided into two eradication groups with a computer-generated random order in the ratio of 1:1 to accept one of the following amoxicillin-containing quadruple regimens for 14 days. Group A received esomeprazole 20 mg twice/day 30 min before meals, amoxicillin 1000 mg twice/day 30 min after meals, colloidal bismuth pectin 200 mg twice/day 30 min after meals, and levofloxacin 500 mg once/day 30 min after meals. Group B received esomeprazole 20 mg twice/day 30 min before meals, amoxicillin 1000 mg twice/day 30 min after meals, colloidal bismuth pectin 200 mg twice/day 30 min after meals, and levofloxacin 200 mg twice/day 30 min after meals.

The medical staff in the gastroenterology unit thoroughly explained the regimen and potential adverse effects to all enrolled patients before therapy. They were also asked to record symptoms of adverse effects during treatment. The patients were given both verbal and written instructions about the importance of taking the medications regularly and advised not to stop the medication in the event of mild to moderate adverse effects. Patients were advised to call the doctors if the side effects were severe. Patients were asked to return within 3 days after *H. pylori* eradication to assess therapeutic compliance and to determine the incidence of adverse effects.

### H. pylori Detection

Patients underwent ^13^C-urea breath tests (UBTs; UCBT Kit, Atom High Tech, Beijing, China) 8 weeks after *H. pylori* eradication to evaluate the therapeutic outcome. Other drugs that could affect the results were prohibited during the study. Antibiotics, bismuth salts, PPIs and histamine-2 receptor blockers were discontinued for at least 4 weeks before the ^13^C–UBT was performed. Patients were instructed to take nothing by mouth 2 hours before the ^13^C-UBT procedure. A baseline breath sample was obtained by blowing through a disposable plastic straw into a 20-mL container, and then, a capsule containing 75 mg of ^13^C-urea was given to patients with 100 mL of water. Another breath sample was collected after 30 minutes. The baseline and 30-minute breath samples were assayed with a mass spectrometer. The test was considered positive if the difference between the baseline sample and the 30-minute sample exceeded 4.0 units.

### Safety and Compliance

Adverse drug reactions and drug regimen compliance were assessed by research staff. Adverse drug reactions were evaluated using open-ended questions in patient self-reports and physical examinations. Adverse drug reactions were classified as mild (not interfering with daily routines), moderate (affecting daily routines), severe (markedly affecting daily routines and discontinued medications), and serious (hospitalization, disability, requiring intervention to prevent permanent damage, or death). The treatment drugs not taken by patients were also counted. Compliance was considered low if less than 80% of the treatment drugs were taken.

### Data Collection

The demographic data and clinical data were collected. The primary endpoint of the study was the eradication rate of *H. pylori*. Secondary endpoints were the rates of adverse drug reactions and the compliance with each regimen. The safety profile (incidence of adverse responses) and compliance (intake of the drugs) were evaluated. *H. pylori* infection was defined by the difference between the baseline sample and the 30-minute sample exceeding 6.0 units. *H. pylori* infection was considered eradicated if the result of the single ^13^C-UBT was negative.

### Statistical Analysis

Our sample size (N = 390) was planned before the study, based on the previously reported eradication rate by the amoxicillin-containing quadruple regimen of approximately 90%. Calculations were based on the assumptions of a = 0.05 and 1–β = 0.80 and an expected between-groups difference of 10% or greater, which was thought to constitute the minimum clinically meaningful difference. Percentages were used to describe categorical variables, and means (standard deviation) or medians (range) were used to describe continuous variables. For categorical variables, associations between 2 groups were evaluated with a chi-squared test (applying Fisher’s correction when necessary), and for continuous variables, the 2-sample t test or the Mann–Whitney U test was used. All statistical analyses were performed using the IBM SPSS 18.0 software. All tests were performed with the a-level set to 0.05 (2-tailed).

## Results

**Figure 1:**
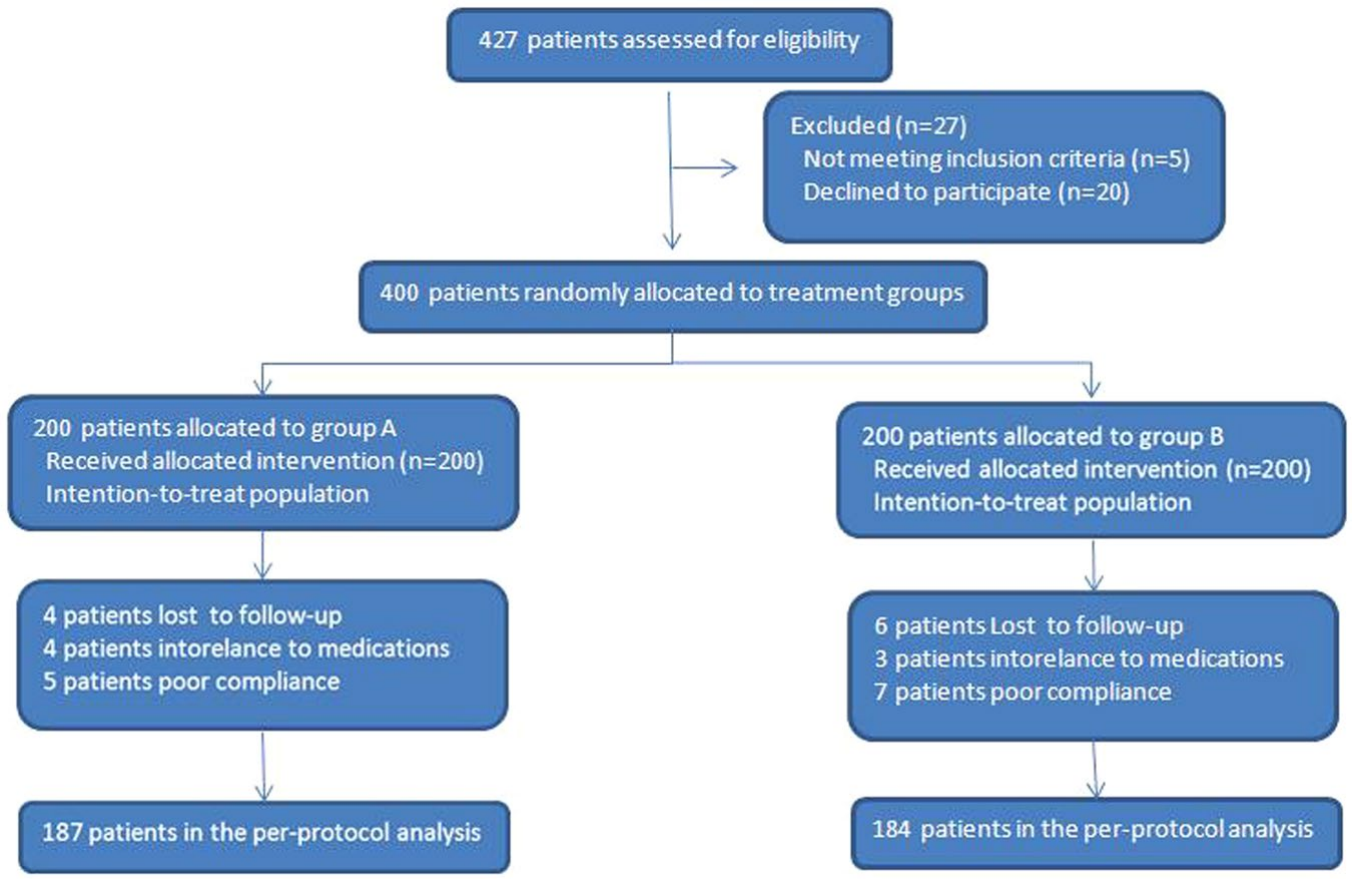
The patients flowchart.

Figure 1 shows the patients flowchart. A total of 427 patients were eligible for enrollment, but 5 were excluded due to a history of allergy to one of the study medicines, and 22 refused to participate in the study. Finally, a total of 400 patients were included and divided into the two eradication groups. The baseline characteristics of the 2 groups were not significantly different. The ages of groups A and B were 40.5 ± 10.5 and 41.4 ± 10.5 years, respectively (mean ± standard deviation, P = 0.392). Men represented 57.5% (n = 115) of group A and 50.0% (n = 100) of group B (P = 0.133) (Table 1). Cigarette smokers represented 18.0% (n = 36) of group A and 13.5% (n = 27) of group B (P = 0.217).

**Table 1:** Baseline data of the patients in the two groups.

Variables	GroupA (n = 200)	Group B (n = 200)	P value
Age (mean ± standard division) (year)	40.5 ± 10.5	41.4 ± 10.5	0.392
Gender (male/female)	115/85	100/100	0.133
Cigarette smoking (yes/no)	36/164	27/173	0.217

### Eradication Rates

Four patients in group A and six patients in group B were lost to follow-up. Four patients in group A and three patients in group B were intolerant to medications. There were five patients in group A and seven patients in group B with poor compliance. The eradication rates in group A and group B were 77.5% (155/200) and 79.5% (159/200) (x^2^ = 0.237, P = 0.626), respectively, in the intention-to-treat (ITT) analysis, and 82.9% (155/187), and 86.4% (159/184) (x^2^ = 0.886, P = 0.346), respectively, in the per protocol (PP) analysis (Table 2).

**Table 2:** Eradication rates.

Analysis	Group A (n = 200)		Group B (n = 200)		P value
	Frequency (n/N)	Eradication rate (%)	Frequency (n/N)	Eradication rate (%)	
ITT	155/200	77.5	159/200	79.5	0.626
PP	155/187	82.9	159/184	86.4	0.346

ITT = intention-to-treat, PP = per protocol.

### Safety and Compliance

There were 45 patients in group A and 49 patients in group B with adverse drug reactions (Table 3). Two patients in group A and one patient in group B discontinued drugs because of serious skin rashes. The incidence and severity of adverse drug reactions, the number of patients who discontinued medicines due to adverse drug reactions, and compliance were not significantly different between the two groups.

**Table 3:** Safety and compliance.

Variable	Group A (n, %)	Group B (n, %)	P value
**Adverse drug reaction**			
*Abdominal pain*	5	7	0.869
*Abdominal distention*	5	5	
*Diarrhea*	11	8	
*Nausea*	3	6	
*Fatigue*	3	3	
*Drowsiness*	1	0	
*Palpitations*	2	4	
*Dizziness*	4	3	
*Skin rash*	6	9	
*Anorexia*	5	4	
**Adverse drug reaction classified**			
*Mild*	31	34	0.794
*Moderate*	12	14	
*Severe*	2	1	
*Serious*	0	0	
Discontinued medicines due to adverse effects	2	1	1.000
Good compliance	195	197	0.724

## Discussion

*H. pylori* is the most common chronic bacterial infection in humans^19^. Bismuth + PPI + amoxicillin + levofloxacin is one of the BQT regimens widely used for the eradication of *H. pylori* infection. Most studies have used oral levofloxacin 500 mg once daily for the eradication of *H. pylori* infection. The recommended dosage of levofloxacin in The Fourth Chinese National Consensus Report on the management of *H. pylori* infection is 500 mg once daily or 200 mg twice daily^20^. P.-Y. Chen *et al*. showed that levofloxacin given once daily appeared to be more effective than when it was given twice daily^21^. However, in this study, we found that the eradication rates with levofloxacin taken as 500 mg once daily or 200 mg twice daily for the cure of *H. pylori* infection were not significantly different (77.5% vs. 79.5%, respectively, P = 0.626).

In our trial, both regimens were simple and safe with few adverse drug reactions. The differences in the incidence of adverse drug reactions and compliance were not statistically significant between the two groups, suggesting that the safety profile and compliance of the patients to both regimens were good. Most of the adverse drug reactions were transient and mild to moderate. The rates of discontinuation of medicines due to intolerance to side effects were not significantly different between the two groups (1.0% vs. 0.5%, respectively, P = 1.000), and no serious adverse drug reactions occurred. The compliance of the patients between two groups was not significantly different (97.5% vs. 98.5%, respectively, P = 0.724). This study suggests that oral levofloxacin 200 mg twice daily was similar in efficacy to oral levofloxacin 500 mg once daily for the eradication of *H. pylori* infection. The cost of treatment may partly affect the decision to select a certain treatment regimen. In economic terms, oral levofloxacin 200 mg twice daily is relatively inexpensive compared with oral levofloxacin 500 mg once daily and is a reasonable treatment for the eradication of *H. pylori* infection.

Bismuth has a synergistic effect with antibiotics and decreases the bacterial load to increase the eradication rate of *H. pylori*. The Maastricht V/Florence Consensus Report recommended that a bismuth-containing quadruple treatment could be used as the first-line treatment in areas with high dual clarithromycin and metronidazole resistance. In the present study, the eradication rate of 2 bismuth-based quadruple regimens was less than 80%, which was lower than the rates reported in other studies^22,23^. The reasons for this lower eradication rate could be as follows: (1) Levofloxacin is readily available in China, and there is a high degree of resistance to it. Those patients with past histories of levofloxacin use, which might have induced drug resistance, were not excluded from the study. (2) Those studies with higher eradication of *H. pylori* infection used other bismuth preparations such as bismuth potassium citrate for the eradication of *H. pylori* infection, but we chose colloidal bismuth pectin in our study. There is no consensus on the dosage of colloidal bismuth pectin that should be used for the eradication of *H. pylori* infection. The dosage of colloidal bismuth pectin used for the eradication of *H. pylori* infection might not have been sufficient in the present study. The optimal dosage of colloidal bismuth pectin that should be used for the eradication of *H. pylori* infection might require more investigation.

The limitations of this study were as follows: (1) This was a single-center study. (2) Most patients enrolled were Han Chinese. There might be ethnic differences in the effectiveness of levofloxacin. Future trials involving multiple centers, regions and ethnicities are warranted to verify the findings. (3) No one underwent susceptibility culture tests before treatment. (4) *H. pylori* stool antigen tests are not widely available in China. A second non-invasive diagnostic method was not used to confirm the *H. pylori* infection.

In conclusion, the results of this study show that oral levofloxacin 200 mg twice daily was similar in efficacy but with lower mean total costs than oral levofloxacin 500 mg once daily when used for the eradication of *H. pylori* infection. It seems reasonable to prefer oral levofloxacin 200 mg twice daily instead of oral levofloxacin 500 mg once daily for the eradication of *H. pylori* infection.
